# An organizational framework and strategic implementation for system-level change to enhance research-based practice: QUERI Series

**DOI:** 10.1186/1748-5908-3-30

**Published:** 2008-05-29

**Authors:** Cheryl B Stetler, Lynn McQueen, John Demakis, Brian S Mittman

**Affiliations:** 1 Independent Consultant, Amherst, Massachusetts, USA; 2 Office of Quality and Performance, U.S. Department of Veterans Affairs, Washington DC, USA; 3 (Retired) Health Services Research and Development Service, U.S. Department of Veterans Affairs, Washington, DC, USA; 4 VA Center for the Study of Healthcare Provider Behavior, Veterans Affairs Greater Los Angeles Healthcare System, Los Angeles, California, USA

## Abstract

**Background:**

The continuing gap between available evidence and current practice in health care reinforces the need for more effective solutions, in particular related to organizational context. Considerable advances have been made within the U.S. Veterans Health Administration (VA) in systematically implementing evidence into practice. These advances have been achieved through a system-level program focused on collaboration and partnerships among policy makers, clinicians, and researchers.

The Quality Enhancement Research Initiative (QUERI) was created to generate research-driven initiatives that directly enhance health care quality within the VA and, simultaneously, contribute to the field of implementation science. This paradigm-shifting effort provided a natural laboratory for exploring organizational change processes. This article describes the underlying change framework and implementation strategy used to operationalize QUERI.

**Strategic approach to organizational change:**

QUERI used an evidence-based organizational framework focused on three contextual elements: 1) cultural norms and values, in this case related to the role of health services researchers in evidence-based quality improvement; 2) capacity, in this case among researchers and key partners to engage in implementation research; 3) and supportive infrastructures to reinforce expectations for change and to sustain new behaviors as part of the norm. As part of a QUERI Series in *Implementation Science*, this article describes the framework's application in an innovative integration of health services research, policy, and clinical care delivery.

**Conclusion:**

QUERI's experience and success provide a case study in organizational change. It demonstrates that progress requires a strategic, systems-based effort. QUERI's evidence-based initiative involved a deliberate cultural shift, requiring ongoing commitment in multiple forms and at multiple levels. VA's commitment to QUERI came in the form of visionary leadership, targeted allocation of resources, infrastructure refinements, innovative peer review and study methods, and direct involvement of key stakeholders. Stakeholders included both those providing and managing clinical care, as well as those producing relevant evidence within the health care system. The organizational framework and related implementation interventions used to achieve contextual change resulted in engaged investigators and enhanced uptake of research knowledge. QUERI's approach and progress provide working hypotheses for others pursuing similar system-wide efforts to routinely achieve evidence-based care.

## Background

The persistent gap between available evidence and current practice in health care reinforces the challenge of finding effective solutions [1,2]. Contributing to this current status have been the complexity of the change process, limitations of research on implementation, and slow recognition of the critical role of organizational context. Recent progress has been made in some of these areas. For example, methodological issues relevant to implementation are increasingly recognized and addressed [3-5]; and clinician and system barriers to the uptake of evidence have been extensively studied, with solutions being suggested and tested [6,7].

Additional progress is needed regarding the role of organizational context. Specifically, system-level strategies and receptive contextual elements required to achieve routine implementation of research need to be identified and validated [8,9]. This article is one in a *Series *of articles documenting implementation science frameworks and approaches developed by the U.S. Department of Veterans Affairs Quality Enhancement Research Initiative (QUERI). QUERI is briefly outlined in Table 1 and described in more detail in previous publications [10,11]. The *Series' *introductory article [12] highlights aspects of QUERI that are related specifically to implementation science, and describes additional types of articles contained in the *Series*.

**Table 1:** The VA Quality Enhancement Research Initiative (QUERI)

The U.S. Department of Veterans Affairs' (VA) Quality Enhancement Research Initiative (QUERI) was launched in 1998. QUERI was designed to harness VA's health services research expertise and resources in an ongoing system-wide effort to improve the performance of the VA healthcare system and, thus, quality of care for veterans.

QUERI researchers collaborate with VA policy and practice leaders, clinicians, and operations staff to implement appropriate evidence-based practices into routine clinical care. They work within distinct disease- or condition-specific QUERI Centers and utilize a standard six-step process:

1) Identify high-risk/high-volume diseases or problems.
2) Identify best practices.
3) Define existing practice patterns and outcomes across the VA and current variation from best practices.
4) Identify and implement interventions to promote best practices.
5) Document that best practices improve outcomes.
6) Document that outcomes are associated with improved health-related quality of life.

Within Step 4, QUERI implementation efforts generally follow a sequence of four phases to enable the refinement and spread of effective and sustainable implementation programs across multiple VA medical centers and clinics. The phases include:

1) Single-site pilot,
2) Small-scale, multi-site implementation trial,
3) Large-scale, multi-region implementation trial, and
4) System-wide rollout.

Researchers employ additional QUERI frameworks and tools, as highlighted in this *Series*, to enhance achievement of each project's quality improvement and implementation science goals.

This *Series *paper addresses the issue of organizational context, specifically in terms of the following:

▪ Organizational elements targeted for change within the VA to enhance use of research as the norm, and

▪ The approaches or implementation strategy used to create and support those contextual changes.

### Evidence-based practice and the context of change

Delivery-focused organizational interventions have been well researched, such as the revision of clinical roles and use of integrated care services [13]. However, little research regarding organizational management interventions exists relative to the routine implementation of evidence-based practices (EBP) [8,9].

A growing body of knowledge does provide related insight into the importance of organizational context to general change [14,15]. Published literature, for example, points to the potential importance of both a receptive context for innovation [8,16] and a strategic approach to – and management of – related organizational innovation and change [17]. More specifically, it has been proposed that "methods and strategies for integrating use of evidence into the fabric of the clinical organization" are needed if an organization wishes to implement EBP as a norm [9]. Since such broad-based change does not usually occur naturally, managerial guidance and strategic management of targeted interventions are often required over a considerable period of time [18].

VA's QUERI program has served as a natural laboratory for exploring both key contextual elements and a strategic approach to implementation in real-time settings. This article describes organizational changes linked to QUERI in terms of an underlying framework and implementation strategy. The changes associated with QUERI's institutionalization occurred within an evolutionary process, which has achieved considerable success and remains focused on an innovative integration of health services research, policy, and clinical care delivery.

### Meeting the challenge of organizational change

In 1998, the VA initiated QUERI to facilitate improvement of health care quality for its patient population (U.S. veterans). QUERI's goal was to improve performance throughout this national health care system through the increased and systematic use of research. The complexity of this task was at first not apparent. Preparatory planning for QUERI had established a guiding, 6-step framework [12] [Table 1] and assigned responsibility to organize the program to VA's headquarters-based Health Services Research & Development Service (HSR&D). Thus, VA's researchers were asked to play a leading role in "purposely link(ing) research activities (which generate scientific evidence) to clinical care in as close to real time as possible, thereby leading to rapid adoption of best clinical practices and improvement in patient outcomes [p. I-14, [19]]."

Preparatory efforts also included creation of QUERI Centers to operationalize expectations [12] and a supportive infrastructure within HSR&D [20]. The infrastructure included various advisory committees with multiple stakeholder members from outside of HSR&D. Such members could link QUERI to additional key stakeholders and to the many changes taking place in the larger VA environment. The latter included implementation of electronic patient records, as well as development and dissemination of both evidence-based guidelines and guideline-based performance measures.

The above decision-making and operational structures were necessary but not sufficient to achieve the envisioned link between research activities and clinical care – or to achieve the related goal of "rapid" uptake of best practices. Within a short time, it was recognized that "QUERI sits at the intersection of clinical practice and research.... a gray area where integration is not always natural [p. I-24, [20]]."

More specificity thus was needed both to understand how to operationalize links between research and practice and to systematically enhance EBP in routine clinical functions. Emerging questions from key stakeholders, particularly QUERI Center researchers, helped to identify both specific barriers to involvement and critical organizational issues. It became apparent that the desired shift to a more consistent, "proactive, interactive and multi-faceted implementation role for health services researchers in the context of a close collaboration between research, quality improvement (QI), and clinical leadership" [12], p 7] was not necessarily a natural fit with the existent culture [21]. To move forward, barriers had to be addressed and proactive organizational actions taken. Such actions had to promote incremental shifts toward new awareness and values; and they also had to focus on related capacities, structures and processes.

The remainder of this article describes, first, a framework that identified and clarified actions needed over time to meet evolving challenges and enhance QUERI's progress. It then provides information on implementation interventions and contextual changes relevant to each of the underlying framework's elements: culture, capacity, and infrastructure. The paper ends with a discussion of QUERI's achievements and the potential applicability of this approach to others' organization-wide change.

### A framework for informing implementation of organizational change

The implementation strategy used to achieve QUERI's present status drew upon the best available evidence regarding organizational change, EBP, and implementation science available in the late 1990s and early 2000s [22-24]. Overall, it is best described within an evidence-based, organizational change framework. This framework had previously served to "re-energize mature" organizations [14,18]. It contains three interacting elements related to institutionalizing an organizational level change: 1) refinement of a culture, 2) capacity-building efforts, and 3) a supportive, reinforcing, and sustaining infrastructure

This framework's premise is that to become a routine part of a system, an innovation such as QUERI needs leadership support for related changes. Such changes likely will involve cultural values and norms, resources and expertise that enable stakeholders' engagement and capabilities, and an alignment of various organizational systems and processes with the innovative Program's requirements.

Over time, implementation interventions targeted at each individual organizational element are assumed to act synergistically with the other elements. When successful, these changes collectively help to create and sustain new norms within the organization. The innovation gradually becomes a part of routine practice across the system, and ongoing, related efforts occur more naturally and are more easily sustained.

### Re-orienting cultural norms and values

Culture is herein defined as an organization's values, norms and expectations – or "the way things are done." Culture and its sub-components are increasingly recognized as a critical factor in implementation of EBP [8,14,25]. One such sub-component is the managerial sub-culture and how supportive and interested managers are in promoting the use of evidence and related changes.

Prior to QUERI, the VA recognized the value of research to improving patient care and had instituted a strong intramural research program designed to enhance the health of its patient population [19]. In 1998, the VA also had a general expectation and value for collaboration. With QUERI, VA leadership expanded these expectations by asking VA researchers to do the following: Engage with policymakers and clinically-based stakeholders as "integral partners" [26,27]; and actively facilitate VA quality improvement through the use of research findings and measurement of impact.

With this shift, clinical stakeholders were no longer merely sources of data and study sites, and the endpoint of research was not simply a report to be used by others. QUERI researchers and those they partnered with at the bedside would, together, focus directly on the real-time needs of decision-makers and clinicians.

As QUERI evolved, VA leadership began to articulate and consistently champion these expectations for desired change. Both QUERI and non-QUERI VA leaders engaged stakeholders in informative and persuasive communications. Early on, leadership established regular communications with QUERI Center Coordinators [12] to clarify and reinforce new expectations and underlying values. For example, QUERI leadership reinforced the congruence of QUERI's mission with the VA's overall mission to "do the right thing for patients." Additionally, QUERI leadership was responsive to feedback regarding challenges confronting QUERI Center researchers and actively facilitated the reduction of obstacles.

QUERI-related communications also engaged clinical leadership, leading to an important early success. Specifically, clinical directors of VA's regional delivery networks, as well as leaders of VA headquarter departments, agreed to allocate clinical funds on a routine basis to facilitate QUERI's activities [12]. This clinical funding is separate from the research funding supporting VA's traditional health services research (HSR) studies. This direct and successful engagement of clinical stakeholders was one of the clearest messages for QUERI Centers of the widespread value for the program within VA.

Communications involve language and views or paradigms of the world, both of which are key aspects of culture. Therefore, leadership took steps to facilitate a greater understanding of QUERI expectations through related tools; in this case, relative to HSR. These tools were developed and adapted over time to encourage researchers across the diverse set of QUERI Centers to use consistent language to discuss implementation of evidence into practice. For example, in 2001 a glossary was designed to enhance communication and a common understanding of implementation and related research terms (see Table 1 in the *Series *Overview article [12]).

Given the difference between traditional forms of research familiar to the majority of VA HSR researchers and the more active, improvement-oriented type of implementation research within QUERI [12], new concepts and detailed explanations also were communicated. This served to enhance awareness of and appreciation for alternative approaches to implementation research. This new paradigm of HSR, unfamiliar to most QUERI investigators, is synopsized in Table 2 and termed "hands-on" implementation research.

**Table 2:** Operational definition of hands-on, action-oriented research on implementation

1. Hands-off implementation research, in contrast to hands-on implementation research, is often demonstrated by the following, i.e., researchers:
i) Allow sites to view the study as "your research;"
ii) Drop intervention/s into the site, then sit back and wait till the end of a trial to see progress and related factors;
iii) Delegate site activities to research assistants that would be critical to routine best practice maintenance after the study;
iv) Plan not to interfere with experimental interventions, or perhaps even explore fidelity or actual implementation (i.e., given need for maximum control); and
v) Are primarily concerned with statistical outcomes re: targeted variables rather than also understanding the complex black box of implementation.

2. Hands-on implementation research includes or is demonstrated by the following actions, i.e., researchers:
i) Engage in a strategic, collaborative relationship; i.e., they initiate a strategic effort to partner with relevant operational leadership by:
▪ Engaging key stakeholders in a mutual relationship regarding improvement needs,
▪ Enhancing partner commitment (as through evidence-based persuasion/gaps evidence, stakeholder needs assessment, and use of a business case); and
▪ Focusing the partner on the fact that this is not "research as usual," but rather a quality improvement effort with a rigorous study and evaluation approach to enable actual improvement and replication in other clinics/sites.


ii) Participate in the implementation process on site, as appropriate, in order to:
▪ Understand, real-time, the ongoing nature of implementation within the particular setting – but not to substitute for roles/activities that will need to be sustained/maintained as part of the routine delivery system or practice; and
▪ Provide formal facilitation to help overcome mutable problems and provide needed support [40].


iii) Utilize a hybrid study design which:**
▪ Involves the most realistically rapid timeline given the complexity of the implementation program,
▪ During the study, focuses on progress and identifies both potential and actual influences on the progress and effectiveness of implementation efforts through the use of formative evaluation [3], and
▪ Plans action during the study, as needed based on formative data, to refine the change intervention, resolve mutable barriers, and enhance available facilitators, in order to optimize:
∘ Actual implementation of the change intervention to achieve or at least assess its potential;
∘ The goal of clinically meaningful, not just statistically significant, evidence-based practice;
∘ Understanding of the black box of implementation, including cost-benefit;
∘ Identification of outstanding research questions; and
∘ Development of a replicable implementation program.


3. Summary: Key words which describe "hands-on" implementation research:
▪ STRATEGIZE
▪ ENGAGE/EDUCATE/PERSUADE
▪ PARTICIPATE
▪ FACILITATE
▪ OPTIMIZE

**A hybrid design combines the use of formative evaluation with an experimental study, quasi-experimental study or other appropriate real world design for the question/targeted innovation at hand, within QUERI's framework, i.e., a continuum of pilot to national rollout phases [12].

In summary, by the end of the second full year of QUERI, several cultural triggers perceived as critical to QUERI's evolution were enacted and reinforced to enhance stakeholders' interest in facilitating EBP. These triggers primarily involved direct leadership actions and visible supportive behaviors that communicated the value of a more active implementation role for QUERI investigators. Specific leadership actions employed as an implementation strategy included role modeling; use of special language; facilitative networking; ongoing, explicit advocacy; and celebration of small wins. VA leadership also provided ongoing encouragement to QUERI Centers, regular contact regarding program expectations and progress, and clarifying documents. Of note is the fact that the desired shift was occurring within the context of a broader, congruent VA transformation, wherein expectations focused on achieving the highest known standards for care. This reinforcing transformation is further described in the Series Overview [12] and had at its core a quality improvement lens.

### Building capacity to engage in an improvement-focused program of research

In addition to increased awareness, clear expectations, a common language and enhanced collaborative connections, those expected to successfully operationalize desired change must have the capacity, capability, and resources to do so [14]. Thus, leadership provided QUERI researchers and key stakeholders (e.g., selected members of advisory committees) with the support needed to obtain knowledge, skills, and opportunities to meet new expectations.

The creation of both new types of funding and new study review mechanisms were instrumental in transitioning from traditional HSR to a more improvement-oriented approach. So too was the availability of various toolkits. QUERI Centers made use of these capacity-building supports to fulfill their operational mission, as described in the *Series *Overview [12]. Such interventions reflect the same types of implementation approaches employed in clinical implementation projects. Examples of this change strategy, based on the best evidence available in the early years of QUERI [23,24] are described below. Briefly, these focus on educational/expert resources, incentives, and a methodological toolkit.

#### Educational programs, reference material and expert resources

QUERI national conferences were held annually in the initial years of the program (and will begin again in 2008). These provided a setting for education and a forum for clinical and operations leadership to clarify program goals and direction. By highlighting current Center efforts, a venue was created for sharing exemplar activity, recognizing common issues requiring additional supports, and identifying common findings and themes across QUERI conditions. Common "lessons learned" and "best practices" could be gleaned through interactions during seminars, workshops, and poster sessions. Tools also were exchanged, and a web-based *Implementation Guide *[28] was found to be one of the more useful tools for those new to QUERI.

Through annual meetings and ongoing dialogue, QUERI Centers accessed an additional resource – the technical assistance and consultation of a few implementation experts. These experts participated in key QUERI committees and were well-informed of Program expectations and resources. They provided consistency of core ideas, which facilitated the education of both investigators and committee members less familiar with implementation science. They also helped to keep leadership up-to-date with emerging science. QUERI's experts were available for individual Center consultations, site visits, and educational programming.

To provide additional and more readily accessible resources, special funding was made available to QUERI Centers. This supported use of short-term local academic experts and, as described in the infrastructure section below, appointment of an implementation research coordinator for each QUERI Center. Such experts came from the fields of organization and management science, nursing, educational psychology, health administration and policy, anthropology, and related fields.

Centers also were offered special data resources, given the various and often complex data processes inherent throughout the QUERI framework. For example, Centers were provided with critical expert resources familiar with VA databases and with health economics – an expected component of implementation studies [29,30], as well as a Data Issues Working Group (DIWG). The DIWG was established as a resource for problem solving. With the DIWG, QUERI Centers could resolve common data issues together rather than each having to address their needs individually. Over time, through such "educational/resource" tools, QUERI Centers moved toward an understanding of key themes as well as consistency and confluence of thoughts and ideas relevant to implementation.

#### Incentives

QUERI expectations for improvement studies [Table 2] were not a perfect fit with the standard HSR&D paradigm and related review structures and processes. Thus, funding opportunities were developed in the form of special study solicitations. Concomitantly, as further incentive to use of more innovative, action-oriented approaches, QUERI created special review committees. These committees included members who were knowledgeable of implementation science and open to relevant designs, concepts and approaches unfamiliar to many current HSR&D reviewers. Committee members used revised criteria relevant to the QUERI model to judge special funding proposals. These criteria are described in the next section under 'Service-Directed Projects' and in the *Series *Overview [12]. [See Additional file 1: 'QUERI Service-Directed Projects: Proposal review criteria.']

Special solicitations enabled Center researchers, eager to start new implementation studies, to more fully meet expectations since funding and review paths were now better aligned with QUERI goals. Such funding included opportunities for collaboration between researchers and clinical operations, with a particular emphasis on using the QUERI 6-step model [12] to improve care delivery. [See Additional file 2: 'Special solicitation for projects implementing research into practice to improve care delivery;' Additional file 3: 'VISN/HSR&D implementation collaborative: Innovations to implement evidence-based clinical practice;' and Additional file 4: 'Special solicitation for Service-Directed Projects (SDP) on implementation of research into practice'].

#### A guiding methodological toolkit

An additional strategy was designed to enhance the capacity of QUERI researchers to deal with the inherent methodological challenge of implementation research. In the early years of QUERI in particular, QUERI researchers faced multiple challenges:

▪ Selection of implementation designs and tools that would address the complexities of producing credible output while accounting for the realities of a real-time, dynamic health care setting [3,31];

▪ Completion of implementation research within a shorter timeframe than the 3–5 year period common for most VA health services research; and

▪ Maintenance of rigor in QUERI's improvement research paradigm.

The related implementation strategy, which evolved over time, centered on innovative documents and toolkits created specifically for the Program. Of special note are the expanded QUERI framework [Table 1] and the Service-Directed Project template [see Additional file 1 and Additional file 4]. In terms of the QUERI framework, the Overview for the *Series *describes its evolution and provides detailed tables outlining its components, including a 4-phase progression or pipeline of implementation studies [Table 1] [12]. Other *Series *papers illustrate its use [32-34].

The SDP template was an innovation that reinforced implementation science and related concepts not well known to many QUERI researchers [22-24]. This template highlighted the importance of intervention mapping, theoretical frameworks, strength of evidence issues, formative evaluation, and spread and sustainability issues, as well as the critical role of context [3,15,35,36]. The SDP template and its separate funding mechanism facilitated innovative exploration and a learning period for VA researchers. More specifically, the template enhanced understanding of new concepts and encouraged the investigation of less familiar methodologies. This included designs that would recognize the hybrid nature of QUERI projects to simultaneously improve care and study implementation. It also included a focus on the following types of study approaches: optimization of implementation by refinement of adoption strategies during a study; modification of changeable barriers rather than only their measurement; focus on clinical as well as statistical outcomes; and use of formative evaluation to better understand the black box of implementation.

In summary, considerable attention was paid to building capacity for action-oriented, collaborative implementation research [12] [Table 2]. However, again, capacity building interventions were necessary but not sufficient to fully achieve and sustain the desired organizational evolution.

### Creating supportive infrastructures to reinforce and sustain new behaviors

Building upon the momentum of a beginning cultural shift and progress in capacity-building, QUERI leadership began to make new expectations and related activities an integral part of the everyday organization [18]. More specifically, to sustain progress in the envisioned direction, leadership began the process of creating a supportive infrastructure. This involved revisions over time in relevant organizational policies, procedures, operational systems, roles, and decision-making structures and processes. In many cases, an incremental approach was taken, beginning for example with suggestions in feedback reports. Progress was then monitored and, as needed, explicit expectations and related supportive structures developed. Examples of such institutionalization relate to formal performance expectations and a special implementation role.

#### Performance expectations and related monitoring

The decentralized structure of QUERI Centers was originally established to operationalize QUERI's vision [12]. These Centers routinely focus on EBP via programs of active, collaborative, scientifically-focused implementation research designed to result in national roll-out and the evaluation of evidence-based practices. Each Center is focused on a high-risk and/or high-volume disease or condition that is prevalent among U.S. veterans, such as substance abuse disorders, diabetes, spinal cord injury, and heart disease.

Very specific expectations, versus the more general expectations initially established for these Centers, evolved over time. One example relates to collaboration. Already a general expectation within VA, QUERI leadership at first encouraged active research-practice collaboration; for example, through recommendations regarding membership composition of each QUERI Center's guiding Executive Committee (EC) [12]. Over time, collaboration-related recommendations were broadened and eventually formalized via yearly performance expectations for each Center. These include expectations that Centers establish explicit critical partnerships across VA organizational lines, especially with clinicians, but also with quality improvement, operations leadership, and other QUERI Centers.

Another example of evolving and now formalized expectations relates to implementation science. From the beginning, QUERI Centers were expected to improve patient care and related outcomes and, simultaneously, contribute knowledge to the field of implementation science. Because additional progress on the latter expectation was needed, QUERI Centers are now to report yearly on specific implementation science goals.

Collection of evidence regarding progress and facilitating/hindering factors was one method employed by QUERI leadership to determine the need for guiding infrastructures. A continuous stream of evidence for that purpose came from the following established processes:

∘ Scientific and goal-oriented critiques by a Research & Methodology [R&M] Review Committee, established to provide guidance to QUERI leadership on program and policy-related issues [20]. Members of this group, as well as QUERI leadership, evaluate a periodic strategic plan and a yearly annual report submitted by each QUERI Center. Many R&M members also are in a position within VA to assess the routine progress of QUERI overall and/or that of individual Centers.

∘ Written summary observations from implementation experts, based on overall strategic plans, annual reports, and interaction with Center coordinators.

∘ Critique by SDP review committees on submitted implementation project proposals and outcomes of funded implementation studies.

∘ Ongoing dialogue between QUERI leadership and Center coordinators [12], including solicited and spontaneous feedback from the latter on progress and needs.

One of the most significant examples of monitoring progress relates to the longevity of a QUERI Center and its leadership. In the beginning or "engagement phase," QUERI leadership used encouraging words, gentle persuasion, championing, and "soft" feedback to off-target responses by various Centers to requests or expectations. Over time, however, leadership established a structured set of time-lined expectations regarding explicit implementation-related activities and products for which QUERI Centers were to be formally held accountable. QUERI Centers must now achieve an acceptable level of performance to maintain funding and their status as a Center. The strategic plan and annual report, built on the 6-step QUERI framework [12], provide the basic job description, related performance expectations, and criteria for periodic renewal of a QUERI Center. New expectations are added as both QUERI Centers and R&M members learn more about the realities and needs of integrated partnerships, implementation science, and institutionalization within a large health care system.

Decisions thus are made periodically as to the level of performance of each Center and, as needed, expectations for improvements are established. When accountability or a "fit" with QUERI goals has not been evident over time, decisions have been made regarding the need for additional reviews, site visits by expert teams, adjusted funding, the Center's leadership, or discontinuation of a QUERI Center (typically with re-competition for a replacement Center).

#### An innovative implementation role

Based in large part on the evaluative data noted above, QUERI leadership determined that Centers needed ongoing, direct assistance with implementation science, particularly in terms of social science concepts and related methodologies. This assistance came in the form of funding for a full-time implementation science expert within each Center. In addition to a Research Coordinator and a Clinical Coordinator, all QUERI Centers were required to have an Implementation Research Coordinator (IRC)[12].

Individuals in this role typically possess a doctoral degree in a social science discipline and may also have formal training in a clinical discipline. QUERI Centers often write their own position description to attract IRC candidates. They do not all see this role in the same way and are given flexibility in finding the best person to suit their needs. For this reason, and the fact that there has been no 'implementation science' specialty in the U.S., there has been significant variation across QUERI Centers in terms of IRC backgrounds. Overall evaluation of what worked and did not work in terms of IRC hiring led to the appreciation that no specific academic preparation or specific experience best fits an "implementation expert." However, cross-Center orientation of new IRCs and QUERI mentoring as needed also are important for success in this role.

The IRC role was designed to facilitate implementation efforts within each Center in general, but quickly became central to the achievement of each Center's implementation science goals. Examples of goals facilitated by IRCs include: exploration of a specific implementation concept, such as facilitation; development or refinement of a methodology, such as formative evaluation or measurement of organizational readiness for EBP; and development or exploration of a conceptual framework for improvement-focused implementation studies. Within a QUERI Center, such activity is not confined to the IRC. However, the existence of that role provides a definitive focus and resource for team members on implementation science.

### QUERI today

QUERI has existed for nearly 10 years and has been continuously funded through VA's research and clinical budgets, indicating lasting, broad-based support. There are now nine QUERI Centers, with additional QUERI Centers under consideration. Each new QUERI Center is more easily oriented to its function and operations given formal role expectations, decision-making structures, collaborative ties, sources of funding, toolkits and criteria for producing QUERI products and outcomes. Numerous such products and outcomes have been generated, often as a result of collaboration with other national VA programs and clinical leadership stakeholders, and many have been widely disseminated. Examples of this progress are provided in Table 3, in other *QUERI Series *articles [12] and related QUERI reports [37-40].

**Table 3:** Examples of QUERI progress

Increased appreciation on the part of researchers for the complexity of sustainable implementation in clinical settings with multiple priorities, and increased appreciation for the knowledge and skill that researchers can bring to the identification and improvement of clinical quality problems.
Identification of numerous gaps in current practice for targeted patient populations/problems, and an implementation portfolio within each QUERI Center focused on those gaps [33,34,39]. Each portfolio is developed within QUERI's 4-phase framework, which consists of an expected sequence of implementation projects from initial feasibility assessment to national roll-out [12].
Adoption of specific performance measures by the Office of Quality and Performance; new policies, such as diabetes eye screening [32]; and the evidence-based removal or addition of targeted medications in the VA's formulary.
Refinement or expansion of several existing VA information technology resources to enhance quality care "by developing entirely new databases and informatics tools, validating and refining existing databases, and analyzing and interpreting their contents [p, 348, [29]]."
Provision of requested research-related information of specific interest to VA leadership or other stakeholders, and facilitation and evaluation of major organizational "best practice" changes under the direction of national clinical specialty leadership [38].
An increased number of publications on a wide range of implementation issues and projects, including papers in peer-reviewed journals on methodological issues and suggested solutions [12].
Beginning evidence of successful implementation, such as increased vaccination rates for spinal cord injury patients, an improved policy for eye care screening in veterans with diabetes, expansion of the number of methadone clinics within VA, and improvement in evidence-based alcohol screening [32,37,43,44,45].

QUERI's implementation expectations pushed cultural boundaries, or, as a recent chief of QUERI noted (personal communication), they "up-ended" the traditional relationship of the health services researcher to the health care system. In the current paradigm, QUERI researchers are a "supplier" of expertise, evidence, and facilitation. Their focus goes beyond "research as usual," leading to implementation projects with direct and immediate meaning for current practice and long-term quality improvement.

When QUERI began in 1998, strategic change within QUERI was very much driven by top leadership, with the aid of the R&M Committee, implementation experts, a belief in a continuous learning process, and the underlying organizational change framework. Various QUERI Centers established since the beginning of the initiative have embraced the new paradigm and are becoming skilled in strategizing and measuring what makes a difference in terms of implementation and targeted improvements. Although Centers vary regarding their implementation focus and interest, their collective success in terms of outcome and process improvements is evident [41-45]. As a result, various Centers serve as a program role model for specific topics, such as national roll-out. In this way, knowledge is shared across the Centers and collective learning is enhanced.

VA's strategic approach to improve performance via the systematic use of research was an incremental, iterative refinement of its organizational context as it expressly related to research-based practice. As a result, there is ongoing expansion of implementation-focused collaboration among central leadership, clinical and operations staff in the field, clinicians, and a large multi-disciplinary group of VA-related researchers [46]. Lessons learned through initial implementation projects with the 4-phase pipeline framework are just now being applied in a new set of projects [12]. This next stage of evolution will likely include further alignment of VA infrastructures, as well as capacity-building efforts geared to ongoing partnerships.

## Summary and conclusion

For those who strive to learn from QUERI's experiences, this strategic effort to embed science into clinical decision-making demonstrates that progress requires concerted, ongoing, strategic, systems-based efforts. Such progress was neither uniform nor neatly predictable; but through a tenacious effort, the initiative led to a shift in cultural norms and values. The organizational framework and related implementation interventions used to achieve contextual change resulted in engaged investigators and enhanced implementation of evidence-based practices.

The transformation described above required resolute, ongoing commitment in multiple forms and at multiple levels. VA's commitment to QUERI came in the form of visionary leadership, allocation of ongoing resources, infrastructure refinements, innovative peer review and study methods, and direct involvement of key stakeholders. Such stakeholders included both those providing and managing clinical care, as well as those producing relevant evidence within a health care system.

Over the past decade, the maturation of QUERI has been complex. Individual-level change has been an inherent part of the process, which sometimes led to discomfort and frustration [21]. Many QUERI researchers needed unfamiliar knowledge and skills. Moreover, they faced frequent and action-oriented reporting tasks, versus the usual end-of-grant-study summary; and ongoing accountability requirements for their performance and for related system needs. They also had to work together in new ways or with new stakeholders, often across varying perspectives.

QUERI's evolution is by no means complete. The nature of the partnerships and arrangements between clinical stakeholders and researchers is still evolving, as described in the current effort to develop a template for evidence-based national roll-out across the VA health care system [46]. However, the momentum is strong, and progress to date illustrates that research and practice can be systematically linked through concerted, system-level implementation efforts that result in measured output and links between interventions and outcomes.

Although most health care systems have not established a dedicated internal research arm, numerous institutions have ongoing relationships with outside investigators, including those interested in measuring quality improvement. They also have leaders who speak about the need for EBP and its value. The success of the strategic approach described in this case study demonstrates its value to the VA. This description of what worked and did not work during QUERI's initial decade also provides insight into how organizations can promote interest in EBP and move it towards institutionalization. The strategic fostering and management of a culture that promotes capacity building and facilitates creation of supportive infrastructures may prove useful to others.

## Competing interests

All authors played central roles in the establishment and/or leadership of QUERI. Dr. Brian Mittman is co-editor in chief of *Implementation Science*. All editorial decisions regarding this manuscript were made by co-editor in chief Dr. Martin Eccles and guest external editor Dr. Ian Graham.

## Authors' contributions

CBS conceived of the paper and drafted the initial form and all revisions of this manuscript. All other authors LM, JGD, BSM read drafted components and contributed to refinement of the manuscript. All authors read and approved the final manuscript.

## Supplementary Material

Additional file 1 QUERI Service-Directed Projects: Proposal review criteria. Review criteria relevant to the QUERI model developed to judge special funding proposals. Click here for file

Additional file 2 Special solicitation for projects implementing research into practice to improve care delivery (2005). A special form of funding and study focus to encourage action-oriented improvement research. Click here for file

Additional file 3 VISN/HSR&D implementation collaborative: Innovations to implement evidence-based clinical practice. A special form of funding and study focus that requires a collaborating partnership between the delivery system and health services researchers to accomplish implementation of EBP and generate knowledge to facilitate spread. Click here for file

Additional file 4 Special solicitation for Service-Directed Projects (SDP) on implementation of research into practice (2003). A special form of funding and study focus to encourage action-oriented improvement research. Click here for file

## References

[B1] McGlynn EA, Asch SM, Adams J, Keesey J, Hicks J, DeCristofaro A, Kerr EA (2003). The quality of health care delivered to adults in the United States. N Engl J Med.

[B2] Shrank WH, Asch SM, Adams J, Setodji C, Kerr EA, Keesey J, Malik S, McGlynn EA (2006). The quality of pharmacologic care for adults in the United States. Med Care.

[B3] Stetler CB, Legro MW, Wallace CM, Bowman C, Guihan M, Hagedorn H, Kimmel B, Sharp ND, Smith JL (2006). The role of formative evaluation in implementation research and the QUERI experience. J Gen Intern Med.

[B4] Grimshaw JM, Thomas RE, MacLennan G, Fraser C, Ramsay CR, Vale L, Whitty P, Eccles MP, Matowe L, Shirran L, Wensing M, Dijkstra R, Donaldson C (2004). Effectiveness and efficiency of guideline dissemination and implementation strategies. Health Technol Assess.

[B5] Glasgow RE, Lichtenstein E, Marcus AC (2003). Why don't we see more translation of health promotion research to practice? Rethinking the efficacy-to-effectiveness transition. Am J Public Health.

[B6] Grol R, Wensing M, Eccles M (2005). The implementation of change in clinical practice.

[B7] Bosch M, van der WT, Wensing M, Grol R (2007). Tailoring quality improvement interventions to identified barriers: a multiple case analysis. J Eval Clin Pract.

[B8] Greenhalgh T, Robert G, Macfarlane F, Bate P, Kyriakidou O (2004). Diffusion of innovations in service organizations: systematic review and recommendations. Milbank Q.

[B9] Stetler CB, Ritchie J, Rycroft-Malone J, Schultz A, Charns M (2007). Improving quality of care through routine, successful implementation of evidence-based practice at the bedside: an organizational case study protocol using the Pettigrew and Whipp model of strategic change. Implement Sci.

[B10] McQueen L, Mittman BS, Demakis JG (2004). Overview of the Veterans Health Administration (VHA) Quality Enhancement Research Initiative (QUERI). J Am Med Inform Assoc.

[B11] Feussner JR, Demakis JG, Kizer KW (2000). VA's Quality Enhancement Research Initiative.. Med Care.

[B12] Stetler CB, Mittman BS, Francis J (2008). Overview of the VA Quality Enhancement Research Initiative (QUERI) and QUERI theme articles: QUERI Series. Implement Sci.

[B13] Wensing M, Wollersheim H, Grol R (2006). Organizational interventions to implement improvements in patient care: a structured review of reviews. Implement Sci.

[B14] Stetler CB (2003). Role of the organization in translating research into evidence-based practice. Outcomes Manag.

[B15] McCormack B, Kitson A, Harvey G, Rycroft-Malone J, Titchen A, Seers K (2002). Getting evidence into practice: the meaning of 'context'. J Adv Nurs.

[B16] Pettigrew A, Ferlie EB, McKee L (1992). Shaping strategic change: Making change in large organizations.

[B17] Pettigrew A (1992). The character and significance of strategy process research.. Strategic Management Journal.

[B18] Stetler CB, Charns M (1995). Collaboration in Health Care: Hartford Hospital's Experience in Changing Management and Practice.

[B19] Feussner JR, Kizer KW, Demakis JG (2000). The Quality Enhancement Research Initiative (QUERI): from evidence to action. Med Care.

[B20] Demakis JG, McQueen L, Kizer KW, Feussner JR (2000). Quality Enhancement Research Initiative (QUERI): A collaboration between research and clinical practice.. Med Care.

[B21] Rubenstein LV, Pugh J (2006). Strategies for promoting organizational and practice change by advancing implementation research. J Gen Intern Med.

[B22] Stetler CB (2001). Updating the Stetler Model of research utilization to facilitate evidence-based practice. Nurs Outlook.

[B23] Rogers EM (1995). Diffusion of Innovations.

[B24] NHS Centre for Reviews and Dissemination (1999). Getting evidence into practice.. Eff Health Care.

[B25] Rycroft-Malone J, Kitson A, Harvey G, McCormack B, Seers K, Titchen A, Estabrooks C (2002). Ingredients for change: revisiting a conceptual framework. Qual Saf Health Care.

[B26] Ross S, Lavis J, Rodriguez C, Woodside J, Denis JL (2003). Partnership experiences: involving decision-makers in the research process. J Health Serv Res Policy.

[B27] Lavis JN, Ross SE, Hurley JE, Hohenadel JM, Stoddart GL, Woodward CA, Abelson J (2002). Examining the role of health services research in public policymaking. Milbank Q.

[B28] QUERI VA (2007). QUERI Implementation Guide. http://www.hsrd.research.va.gov/queri/implementation/default.cfm.

[B29] Hynes DM, Perrin RA, Rappaport S, Stevens JM, Demakis JG (2004). Informatics resources to support health care quality improvement in the veterans health administration. J Am Med Inform Assoc.

[B30] (2006). VA HSR&D Resource Centers. http://www.hsrd.research.va.gov/about/centers/resource_centers.cfm.

[B31] Ovretveit J, Gustafson D (2003). Using research to inform quality programmes. BMJ.

[B32] Krein SL, Bernstein SJ, Fletcher CE, Makki F, Goldzweig CL, Watts B, Vijan S, Hayward RA (2008). Improving eye care for veterans with diabetes: An example of using the QUERI steps to move from evidence to implementation: QUERI Series. Implement Sci.

[B33] Goetz MB, Bowman C, Hoang T, Anaya H, Osborn T, Gifford AL, Asch SM (2008). Implementing and evaluating a regional strategy to improve testing rates in VA patients at risk for HIV, utilizing the QUERI process as a guiding framework: QUERI Series. Implement Sci.

[B34] Brown AH, Cohen AN, Chinman MJ, Kessler C, Young AS (2008). EQUIP: Implementing chronic care principles and applying formative evaluation methods to improve care for schizophrenia: QUERI Series.. Implement Sci.

[B35] Bartholomew LK, Parcel GS, Kok G (1998). Intervention mapping: a process for developing theory- and evidence-based health education programs. Health Educ Behav.

[B36] Patton MQ (1997). Utilization-Focused Evaluation: The New Century Text.

[B37] Francis J, Perlin JB (2006). Improving performance through knowledge translation in the Veterans Health Administration. J Contin Educ Health Prof.

[B38] QUERI VA (2007). Ischemic Heart Disease QUERI. http://www1.va.gov/PS_IHDQUERI/.

[B39] QUERI VA (2007). QUERI's Clinical and Co-Chairs.. http://www.hsrd.research.va.gov/queri/program.cfm.

[B40] Stetler CB, Legro MW, Rycroft-Malone J, Bowman C, Curran G, Guihan M, Hagedorn H, Pineros S, Wallace CM (2006). Role of "external facilitation" in implementation of research findings: a qualitative evaluation of facilitation experiences in the Veterans Health Administration. Implement Sci.

[B41] Curran GM, Mukherjee S, Allee E, Owen RR (2008). A process for developing an implementation intervention: QUERI Series. Implement Sci.

[B42] Weaver FM, Smith B, LaVela S, Wallace C, Evans CT, Hammond M, Goldstein B (2007). Interventions to increase influenza vaccination rates in veterans with spinal cord injuries and disorders. J Spinal Cord Med.

[B43] Bowman CC, Sobo EJ, Asch SM, Gifford AL (2008). Measuring persistence of implementation: QUERI Series. Implement Sci.

[B44] Willenbring ML, Hagedorn H, Postier AC, Kenny M (2003). The opiate agonist therapy effectiveness initiative: Improving treatment for opioid dependence. Poster Presented at the Veterans Affairs' Health Services Research and Development, National Conference.

[B45] Bradley KA, Williams EC, Achtmeyer CE, Volpp B, Collins BJ, Kivlahan DR (2006). Implementation of evidence-based alcohol screening in the Veterans Health Administration. Am J Manag Care.

[B46] Owen RR, Rubenstein LV, Chaney EF, Smith JL (2007). Bringing Evidence-Based Practices into Regional and National Use: The ReTIDES Example.. Workshop at the Veterans Affairs' Health Services Research and Development, National Conference.

