# Supplementary material for: An organizational framework and strategic implementation for system-level change to enhance research-based practice: QUERI Series
Source: Implement Sci. 2008 May 29;3:30. doi: 10.1186/1748-5908-3-30 (PMC2430586; doi:10.1186/1748-5908-3-30)
Supplement: Additional file 1 — QUERI Service-Directed Projects: Proposal review criteria. Review criteria relevant to the QUERI model developed to judge special funding proposals. [file 1748-5908-3-30-S1.pdf]

VA QUERI SERVICE DIRECTED PROJECTS: PROPOSAL REVIEW CRITERIA[1,2]

Adequacy of Response to Previous Reviewer Comments

- ❑ **Did the applicant address the issues raised by the reviewers of the concept paper or previously reviewed full proposal (if this is a resubmission)?**

Project Goal(s) and Questions/Hypotheses

- ❑ **Is the project's overall goal(s) described in light of the quality enhancement (QE) or performance problem(s) the project is targeting?**
- ❑ **If the project aims to address an intervening barrier or factor *contributing* to the quality problem (but not *directly causing* the quality problem), does the proposal explain how the project will contribute to the solution of the overarching quality problem, and why a direct solution is not possible?**

Background of Context

- ❑ **Does the proposal provide an adequate literature review and evidence-based clinical recommendations/guidelines or other foundations supporting the hypothesized effectiveness of the proposed quality improvement approach?**
- ❑ **Is there an adequate description of current practices, determinants, barriers and facilitators?**

Significance

- ❑ **Does the proposal adequately describe the clinical/quality issue(s) to be addressed, including as appropriate data on the clinical condition's/problem's prevalence/incidence, mortality/morbidity, quality of life consequences, economic consequences, or other significant considerations.**
- ❑ **Is the proposed work grounded in theoretical and empirical evidence on organizational change and/or provider behavior?**
- ❑ Will the proposed work contribute to policy, practice and/or the science of Implementation?
- ❑ Will lessons learned from the proposed project be generalizable to other Implementation efforts?
- ❑ **Are the specific research questions/hypotheses clearly stated and appropriate?**
- ❑ Is the project aimed at creating a learning organization focused on the translation of research into practice?

Methods

- ❑ **Is an overall conceptual framework for the approach provided, citing specific sources and justifying the selection of the source(s) and framework for the specific quality problem and intervention approach planned?**
- ❑ **Are the design and methods appropriate given the stated project goals?**
- ❑ **Does the work involve a clearly articulated process or formative evaluation?**
- ❑ **If an intervention is being implemented, is it adequately described (e.g., are components specified, is it apparent who will administer the intervention and to whom it is targeted) and justified?**
- ❑ If applicable, are intervention components described that will perform the functions listed below?
  - Communicate the legitimacy (e.g., evidence base) of recommended practices (to facilitate their acceptance by the target clinicians, managers, patients, and/or other stakeholders),
  - motivate clinician, manager, patient willingness to change via presentation of evidence of a quality/performance problem or via other means

---

[1] See the following for a shortened table. Cheryl B. Stetler, Brian S. Mittman, Joseph Francis (Guest Editors). QUERI SERIES: Overview of the VA Quality Enhancement Research Initiative (QUERI) and QUERI Article Series. Implementation Sci

[2] From: Cheryl B. Stetler, Lynn McQueen, John Demakis, Brian Mittman. QUERI Series: An evidence-based implementation program in the US Department of Veterans Affairs: The role of context and the strategic management of change Implementation Sci

  - establish, disseminate and reinforce professional (patient) norms favoring the recommended practices and countering current (non-adherent) practices and conveying the advantages of the preferred practices over current practices,
  - create or strengthen external expectations and interest in improved quality (among professional, policy, public, special interest, and/or other groups),
  - educate clinicians, staff, patients regarding desired/expected roles, practices and professional behaviors,
  - enhance clinician/staff/patient knowledge regarding the desired clinical practices and/or enhance skills in performing desired practices,
  - create conditions facilitating and favoring desired practices in routine care, including financial, administrative and other conditions,
  - redesign other aspects of the delivery system and organization to facilitate improved practices,
  - implement the desired care model or organizational arrangements and the behavior/organizational change efforts in a manner adapted to the target practice settings, to maximize success,
  - monitor and continually refine implementation of the new practices, including actions needed to remove any barriers that may arise.
- ❑ **Is the overall research design, including issues such as the experimental unit (facility, clinic, team, clinician, or patient) and other major design features justified?**
- ❑ **Are the variables, measures and data collection methods/plans adequately described?**
- ❑ If relevant, is detailed information on methods to study current practice patterns and their determinants provided?
- ❑ If applicable, is a description provided of plans for monitoring implementation and progress of the quality enhancement effort, and refining the effort based upon this ongoing monitoring?
- ❑ Are decisions to attend to certain features of the implementation effort but not others, or to exclude a formative evaluation altogether justified?
- ❑ **Impact (summative) evaluation: overall plan.** Are plans for measuring the quality enhancement program's impacts on key structures, processes and outcomes of interest addressed?
- ❑ **Are plans for identifying and recruiting all relevant participants, including clinicians, other staff (managers, support staff), patients, patient family members or caregivers, etc., discussed and any human subjects issues addressed?**
- ❑ Are the participating project sites, site recruitment processes, and timeline adequately discussed and justified? Listed below are some elements that reviewers may wish to consider:
  - Randomization protocol
  - Usual care condition
  - Assurance of patient safety
  - Sustainability
- ❑ Are the general analytical approaches for quantitative and qualitative data to be collected as part of the diagnostic analysis, formative evaluation and impact/summative evaluation appropriate?
- ❑ **Is an Economic Analysis appropriate to VA decision makers (e.g., cost consequences, as opposed to traditional cost-effectiveness) included?**

<u>Project Organization and Management</u>

- ❑ **Are the investigators clearly qualified to lead the proposed project?**
- ❑ **Is there a sound plan for project management and leadership?**
- ❑ Are the necessary team members identified and are their qualifications, skills, and expertise appropriate for their designated roles within the project?
- ❑ **Does the project team include individuals with expertise in organizational change and management research?**
- ❑ Is it clear that the investigators have access to the data required for successful completion of the proposed work?
- ❑ If existing databases are to be used, is evidence of familiarity with these databases (and awareness of their idiosyncrasies and limitations) included in the proposal?

__Adequacy of Evidence-Base Supporting Implementation *at This Time*__

- ❑ **Does the proposal clearly demonstrate the existence of an adequate evidence-base, in the form of published research and/or guidelines, to warrant Implementation at this time?**
- ❑ Are there known or potential risks to patients if implementation is __delayed__?
- ❑ Is it clear that implementation is not being rushed (therein creating potential patient risk)?
- ❑ Is there a critical level of need and/or urgency for implementation at this time?
- ❑ Does the implementation plan appear to target a viable system or organization (e.g., it may only be feasible to implement an intervention at a single unit – with VISN support – as a *step* toward implementing VAMC- and/or VISN-wide)?
- ❑ Is there sufficient supplemental evidence to support implementation if a substantial body of effectiveness data has not as yet been published (e.g., cumulative efficacy data, practical/clinical evidence, etc.)?

__Involvement of Key Stakeholders__

- ❑ **Is there evidence of commitment, including tangible resources, at all necessary levels (e.g., provider, unit, facility, VISN)?**
- ❑ Are the needs of veterans clearly represented in the project plan either by direct or indirect veteran involvement in process evaluation?
- ❑ Is there a plan for continued feedback from all stakeholders?

__Contribution to the Veterans Health Administration__

- ❑ **Does the proposed work have the clear potential to improve the quality, effectiveness and efficiency of health care in VA and the health status of veterans?**
- ❑ Does the proposed work hold the promise of rapid clinical and organizational improvement?
- ❑ **Is there evidence that the activities planned for implementation would be __sustainable__ beyond the life of the proposed project?**
- ❑ Is there potential for expansion throughout VHA if the implementation is successful at the level proposed within the scope of the project (e.g., if implementation is to take pace within a single unit, is there potential for expansion throughout on or more VISN(s))?

__Budget Efficiency__

- ❑ Is the budget well justified?
- ❑ **Does the budget reflect VISN support (e.g., in-kind support, equipment, facilities)?**

__Evaluation Plan__

- ❑ **Does the proposal include a well-structured evaluation plan?**
- ❑ Is the evaluation plan unbiased and appropriately tailored to the goals of the proposed work?
- ❑ Will mechanisms be in place to continue to track the success of the implementation efforts after the funding period?
- ❑ **Will spread and rollout (i.e., to other sites, clinics, VISNs) be tracked?**

__Dissemination / Implementation Plan__

- ❑ **Evaluate how and when research results will be disseminated and implemented**
