# Supplementary material for: An organizational framework and strategic implementation for system-level change to enhance research-based practice: QUERI Series
Source: Implement Sci. 2008 May 29;3:30. doi: 10.1186/1748-5908-3-30 (PMC2430586; doi:10.1186/1748-5908-3-30)
Supplement: Additional file 2 — Special solicitation for projects implementing research into practice to improve care delivery (2005). A special form of funding and study focus to encourage action-oriented improvement research. [file 1748-5908-3-30-S2.pdf]

DEPARTMENT OF VETERANS AFFAIRS
Veterans Health Administration
Washington DC 20420

October 1, 2005 In Reply Refer To: 124Q

OFFICE OF RESEARCH AND DEVELOPMENT PROGRAM ANNOUNCEMENT:

Quality Enhancement Research Initiative (QUERI)

Special Solicitation for Projects Implementing Research into Practice to Improve Care Delivery

1. Purpose

The mission of the Veterans Health Administration (VHA) is to honor America's veterans by providing exceptional health care that improves their health and well-being. Research has a strategic role within VHA, focusing on clinical and system improvements designed to enhance the well-being of veterans. VHA seeks to increase collaboration between Research and Operations with a particular emphasis on using the QUERI model to improve care delivery. Proposals responsive to this solicitation will include efforts that:

a. Implement research findings and guidelines into routine practice.
b. Evaluate specific programs and strategies to improve VHA health care quality.
c. Develop evidence and insights about effective implementation and quality improvement approaches.
d. Create - through partnerships and collaborations with VHA leaders, managers, and policymakers at local, network, and national levels -

sustainable tools, processes, and infrastructure that support improved quality.

2. Background

VHA is devoting significant effort and resources to improve the quality and outcomes of its health care services. The Quality Enhancement Research Initiative (QUERI) promotes the systematic use of evidence as the basis of clinical decision-making to optimize patient outcomes and achieve ongoing system-wide quality enhancement. For details about the QUERI process and recommended methods for implementation research, see www.hsrd.research.va.gov/queri/implementation.

The QUERI process has been applied to high-risk and/or highly prevalent diseases or conditions among veterans. Currently there are QUERI groups addressing Chronic Heart Failure, Colorectal Cancer, Diabetes, HIV/AIDS, Ischemic Heart Disease, Mental Health, Spinal Cord Injury, Stroke, Substance Use Disorders and Polytrauma and Blast-related Injury. QUERI Centers include a collaborative group of health services and clinical investigators and VA operational leadership, in order to create a framework for national spread and sustainability. The QUERI Program is evolving away from an exclusively disease-specific orientation toward a broader scope of work that addresses improving care for patients with co-morbid conditions, creates durable partnerships between researchers and system leaders, and expands opportunities for researchers to participate in and evaluate the process of national implementation.

3. Coordination with VHA Leadership and Field

Projects responsive to this solicitation are recognized to be at the intersection of clinical practice, quality improvement, and traditional health services research. Projects typically require interaction with VHA Central Office Programs, Veterans Integrated Service Networks (VISNs), or VHA facilities and may include developing plans and mechanisms for the quality improvement activity to be undertaken by VISNs or health systems. Projects should demonstrate the early involvement of system leaders, managers, and policymakers in identifying key problems/questions to

be addressed and designing evidence-based interventions that can be spread throughout VA and sustained beyond the lifespan of the project.

4. Project Features

This solicitation encourages comprehensive Service-Directed Projects (SDPs) that propose to implement improvement programs and evaluate their impact, as well as Rapid Response Projects (RRPs) that address particular focused needs in response to identified performance gaps. Specific instructions and recommended components for each type of proposal are described in the Appendices.

5. Eligibility

In order to apply, principal investigators must meet VA eligibility requirements including holding a VA appointment of at least 5/8 time. While this solicitation is open to all eligible VA investigators, we strongly encourage applicants to explore collaboration and /or coordination with ongoing QUERI initiatives. Contact information for QUERI is available at www.hsrd.research.va.gov/research/queri.

6. Funding

In planning project budgets, applicants are reminded to adhere to Office of Research and Development (ORD) guidelines regarding allowable use of funds for specific categories of expenses. Because implementation projects are funded with Medical Care (870) dollars, no carryover is allowed across fiscal years.

Service Directed Projects (SDPs): Investigators should indicate the estimated study budget and duration in their concept paper (described below). Projects should be designed to produce useful findings in as short a timeframe as possible, including intermediate products (e.g., organizational assessments, gap analyses, toolkits for improvement) that can be shared with VA leaders prior to study conclusion. Investigators should adhere to budget guidelines for Investigator-Initiated Research projects funded by HSR&D. Note that a total budget exceeding $900,000, or duration exceeding three years, will be approved only under exceptional circumstances.

Rapid Response Projects (RRPs): Because RRPs are issued in response to specific requests from ORD, investigators should carefully review the announcement for additional guidance. Most RRP announcements will have a maximum budget of $50,000 and a duration under one year.

7. Funding Decision

Implementation proposals (SDPs and RRPs) will be reviewed for the strength of the underlying evidence base, relevance to the veteran population, relevance to VHA operations, capability of applicant to meet intended outcome of proposed project, and potential contribution of proposed project to the field of implementation science and quality of life to veterans. Additional criteria for evaluation and review vary according to the type of study, but typically include:

a. Known gaps in performance within VHA,
b. Knowledge of the setting and context in which the performance gap occurs (e.g., needs assessment),
c. Presence of a needs or barrier assessment that determines the appropriate targets (e.g., patient, provider, micro-system, or macro-system) for intervention,
d. Feasibility and potential sustainability of the intervention,
e. Inclusion of methods, designs, and approaches (including quasi-experimental designs, qualitative methods, and formative and process evaluations) appropriate to the problem or issue,
f. An appropriate theoretical framework or model for system or organizational change,
g. Experience in using appropriate VHA information systems and data repositories, as well as knowledge of barriers, workarounds, and data quality issues,
h. Collaborations with system leaders, and
i. An evaluation component that includes an economic or business-case analysis to inform future decisions by VHA leaders (see www.hsrd.research.va.gov/queri/economic-analysis.doc).

As with all VA research, funding is contingent upon availability of funds.

8. Format and Submission Instructions

SDP Concept Paper: The SDP application process requires submission of a Concept Paper describing the performance gap, evidence base, proposed intervention, research and evaluation design, key personnel, and estimated duration and budget. Concept papers should follow the format described in Appendix A. Concept papers will undergo a preliminary review by staff in ORD, PCS, OQP, and 10N to determine appropriateness for the SDP funding mechanism and fit with VA priorities. The full SDP proposal should be submitted no later than 6 months after approval of the Concept Paper, and should address the comments, concerns, and suggestions made in the preliminary review.

SDP Applications: The recommended SDP proposal format and instructions for proposal submission are the same as described in the VHA Handbook for Investigator Initiated Research (IIR). An overview of the application process and submission for IIR funding is available in the VHA Handbook on the web at http://www.va.gov/resdev/directive/1204-1hk.pdf and from the Research and Development Office at each facility.

RRP Applications: The RRP proposal format is abbreviated (see Appendix B) and uses electronic submission (via VA email) for most elements. RRPs are submitted in response to specific requests for implementation or quality improvement studies. The subject line of the email should indicate the title of the specific announcement and the name of the principal investigator.

9. Compliance and Reporting Requirements

Implementation projects are subject to the same human subject and privacy protections requirements as other ORD-funded projects. Because implementation projects address issues that often have great urgency, investigators are encouraged to initiate the compliance process as soon as possible (in contrast to the “just-in-time” approach that applies to traditional research studies). As with all research involving

human subjects, funds are not disbursed until ORD receives documentation of IRB approval or waiver. Implementation projects frequently require access to protected health information in the electronic medical record (in which case approval by an IRB or privacy board is required), or to specially created limited data sets (in which case an appropriate Data Usage Agreement may be required by the data owner). Further information about privacy requirements may be found at http://www.virec.research.med.va.gov/References/VirecInsights/Insights-v04n2.pdf

SDPs are subject to the same reporting requirements of other studies managed by HSR&D, including the submission of a final project report. RRPs will require specific products and reports described in the special announcement. Both SDPs and RRPs should be included in the annual reports of Centers of Excellence and QUERI Coordinating Centers according to their specific reporting requirements.

Because Medical Care (870) dollars cannot be carried over into subsequent fiscal years, changes to the protocol, budget, or participating sites that would require modification requests, as well as unanticipated delays (e.g., IRB delays) should be reported as soon as identified.

10. Inquiries

Specific guidance regarding SDP proposal development may be obtained from Linda McIvor, Program Manager QUERI and Service Directed Projects, at Linda.McIvor@va.gov. Inquiries regarding RRP proposals should be directed to Rachael Evans, Health Science Specialist, at Rachael.Evans@va.gov.

11. Due Date

SDP Proposals: An approved concept paper is required prior to submission of a full SDP proposal. Concept papers are accepted on an ongoing basis; please allow 4 weeks for review and feedback. Full proposals are reviewed quarterly. The due dates for SDP proposal submission are: March 1, June 1, September 1, and December 1.

Rapid Response Project Proposals: Requests for RRPs targeting a specific problem or issue will be distributed throughout the VA research community throughout the year. In most cases, the submission date will be two weeks following the date of issuance.

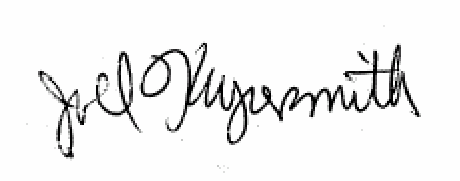

Joel Kupersmith, MD
Chief Research and Development Officer

APPENDIX A
INSTRUCTIONS FOR SUBMISSION OF SERVICE DIRECTED PROJECT CONCEPT PAPERS

The Concept Paper should be 2-5 pages in length (1.5 line spaced or double spaced, exclusive of references).

The investigator should:

1. Summarize the main objectives and specific clinical focus of the proposed project. Briefly describe (1) the clinical/quality issue(s) to be addressed (citing, as appropriate, data on the clinical condition's/problem's prevalence/incidence, mortality/morbidity, quality of life consequences, economic consequences, etc.) and(2) the evidence-based clinical recommendations/guidelines or other foundations for the improvement initiative.
2. Describe the proposed quality improvement strategy or program, citing appropriate literature and evidence supporting the hypothesized effectiveness of the proposed quality improvement approach.
3. Describe the intervention/evaluation design and methods to be used and the type(s) of analyses to be performed.
4. Describe existing or anticipated partnerships with VA operations or program offices.
5. List key personnel involved in the project, including key staff names, affiliations and the discipline or specialty of the P1 and co-PI (if applicable) and other key project participants. Describe the P1 and other project participants' past or current involvement in QUERI (e.g., member of a QUERI Coordinator Center or Executive Committee). Note: HSR&D encourages designation of a single P1; no project may have more than one co-Pl.
6. State the expected project duration and estimated total cost.

Review Criteria

1. The Principal Investigator must be at least a 5/8 VA employee and eligible to conduct research in VA.
2. Concept papers will undergo a preliminary review by staff in ORD, PCS, OQP, and 10N to determine appropriateness for the SDP funding mechanism and fit with VA priorities.
3. The full SDP proposal should be submitted no later than 6 months after approval of the Concept Paper, and should address the comments, concerns, and suggestions made in the preliminary review.

Transmission to QUERI Program

The Principal Investigator should send electronic copies of the Concept Paper to: Linda.Mclvor@va.gov.

Proposal and Concept Paper Submission Dates

The deadlines for full proposals in response to this solicitation are COB March 1, June 1, September 1, and December 1. If a deadline falls on a weekend or holiday, the deadline is the first business day following the deadline. Principal Investigators should notify Linda McIvor at Linda.McIvor@va.gov of Intent to Submit a proposal one month in advance of the submission deadline. Concept papers are accepted on an ongoing basis. Concept paper feedback and notification of acceptance will be provided approximately four weeks after receipt.

Contact Information

Please contact Linda McIvor (Linda.Mclvor@va.gov), Program Manager, QUERI and Service Directed Projects with any questions regarding concept paper instructions or submission dates.

APPENDIX B
INSTRUCTIONS FOR SUBMISSION OF APPLICATIONS FOR RAPID RESPONSE PROJECTS

Implementation Rapid Response Projects (RRPs) are submitted in response to specific solicitations that will be emailed to the appropriate target audience. RRPs can include small pilots or demonstrations, pre-implementation assessments or planning efforts, and studies that follow-up on recently completed implementation activities. Projects may also support development and initial testing of improvement tools, such as registries, clinician decision support tools, or education materials for patients and clinicians.

Application Instructions

1. The Principal Investigator must be at least a 5/8 VA employee and eligible to conduct research in VA.
2. Applicants should submit a plan **no longer than 2 pages** describing**:**
   a. How the project team will complete the work necessary to respond appropriately to the solicitation, and further support the work of ORD's Implementation efforts.
   b. Capacity to complete this work in a timely and high quality manner, as determined by availability of key personnel. Note: the total timeframe for the proposed project may **not exceed 6 months.**
3. All submitted materials not on forms must have margins of at least 1 inch on all sides, and be single-spaced.
4. Each submission must include a face page and an abstract page on forms 10-1313-1 and 10-1313-2.
5. Each submission may include one biographical sketch for each key personnel. Each biographical sketch is limited to 2 pages per person using forms 10-1313-5 and 10-1313-6 or 10-1313-5/6.
6. Please submit budgets on VA forms 10-1313-3 and 10-1313-4. Note that if the project crosses fiscal years, each fiscal year should have its own budget pages. **Total funding cannot exceed $50,000.**
7. No additional attachments will be accepted.
8. **All applications must be received via electronic mail by the due date indicated in the specific solicitation, in one single MS WORD or PDF file. Paper submissions, or submissions sent in multiple files will not be reviewed.**
9. ORD research forms can be downloaded at: http://www.va.gov/resdev/funding/process/forms.cfm

Review Criteria

Applications will undergo peer review by an ad hoc committee. The research team will be notified of the funding decision within one month of submission.

To be eligible for this solicitation, projects must:

1. Impact quality and/or outcomes in one or more VA health care facilities, via implementation of evidence-based clinical recommendations or practices
2. Include formative and summative evaluation designed to identify barriers and facilitators to change. Where appropriate, there should be sufficient cost analysis to inform future resource allocation decisions (http://www.hsrd.research.va.gov/queri/economic-analysis.doc)

3. Involve active partnership of researchers with clinicians, managers, and leaders at VAMC, VISN, or VACO levels

Contact Information

For questions:

Rachael Evans, MPA
202-254-0133
Rachael.Evans@va.gov
