# Supplementary material for: An organizational framework and strategic implementation for system-level change to enhance research-based practice: QUERI Series
Source: Implement Sci. 2008 May 29;3:30. doi: 10.1186/1748-5908-3-30 (PMC2430586; doi:10.1186/1748-5908-3-30)
Supplement: Additional file 3 — VISN/HSR&D implementation collaborative: Innovations to implement evidence-based clinical practice. A special form of funding and study focus that requires a collaborating partnership between the delivery system and health services researchers to accomplish implementation of EBP and generate knowledge to facilitate spread. [file 1748-5908-3-30-S3.pdf]

DRAFT: October 22, 2003

Office of Research and Development
VA Health Services Research and Development (HSR&D)
Targeted Research Announcement

VISN/ HSR&D Implementation Collaborative: Innovations To Implement Evidence-Based Clinical Practice

1. Purpose

The Veterans Health Administration (VHA) Health Services Research and Development (HSR&D) invites applications for collaboration with Veterans Integrated Service Networks (VISN) on a) implementing and evaluating an evidenced-based intervention or b) undergoing and evaluating an organizational or structural change to transform the VISN into a learning organization[1] that can efficiently implement evidence-based practices. Collaborations are intended to help improve clinical services locally within participating VISNs *and* provide templates for expanding successful changes nationwide.

2. Background

VHA needs to develop efficient ways to broadly implement evidence-based practices and foster a learning organization culture that systematically and continuously applies research to improve VA healthcare. Continuous quality improvement (CQI) methods strive to evaluate and improve quality as an ongoing organizational process, but have usually not included an evidence-based intervention focus. Organizational research points to the importance of leadership, teamwork, cultural openness to innovation, and flexible management structures and styles. It also emphasizes integration of quality feedback and data as important organizational characteristics that can improve quality. While these characteristics might help support evidence-based quality improvement, well-defined methods for producing such cultures in clinical organizations do not exist.

3. Objectives for Projects

This solicitation requires a collaborating partnership of VISNs and health services researchers to accomplish two goals:

A) *Facilitate implementation of evidence-based practices.* This facilitation could occur in two ways. First, a VISN could directly implement evidence-based practices. These evidence-based practices should improve care delivery for important clinical areas (e.g. high cost, high prevalence, high need, vulnerable populations). Alternatively, the VISN could undertake organizational or infrastructure development efforts that support evidence based practice

[1] Peter M. Senge et. al., The Dance of Change: the Challenges of Sustaining Momentum in Learning Organizations (New York: Currency/Doubleday, 1999) and The Fifth Discipline Fieldbook: strategies and tools for building a learning organization (New York: Currency, 1994).

implementation (e.g. development of training programs, researching new quality improvement organizational structures).

B) *Generate knowledge that will facilitate implementation of evidence-based practices nationally*. Each applicant team will be responsible for ensuring that progress or findings in its network can be generalized and transformed into useful guidance for other VISNs. This goal will be accomplished by activities that test or develop theories about implementation, and a dissemination plan that ensures the general lessons about implementation, and the specific lessons of implementing individual evidence-based practices, are effectively communicated to other VISNs.

4. Eligibility

The application should originate jointly from a VISN Director or senior Network- Officer (e.g. a Network Clinical Manager) and a collaborating VA researcher. Multiple VISN directors can also collaborate with a single researcher to submit a unified application for multi-network projects. The VISN leader and the researcher should be CO-Principal Investigators, with either listed as “lead”.

5. Finding Collaborators

The new Office of Research and Development Implementation Unit within HSR&D will assist researchers and networks in identifying potential collaborators. Interested researchers without partners should submit an Implementation Topic one page summary. Please prepare the one page summary, in 12 point font, addressing the following areas:

1. PI Contact information (Name, Phone, Email, address)
2. Summary of proposed research (1 or 2 sentences)
3. Purpose/Goals of proposed program
4. Brief description: How this study will facilitate implementation of evidence-based practices and/or generate knowledge that will facilitate implementation of evidence-based practices nationally.
5. Special population or facility needs (if any) from a partner VISN (e.g. a high volume of patients that have a history of homelessness, rural hospitals, a spinal cord injury treatment center, etc.)

One-pagers may be submitted at any time. However, to facilitate partnering in time for the first application, please submit electronically your one-pager by October 24, 2003 (distributed to VISNs on October 27, 2003) or by October 31, 2003 (distributed to VISNs on November 3, 2003) to: Pauline Sieverding, PhD, JD, MPA (pauline.sieverding@hq.med.va.gov). This process is optional. Researchers and Networks also are encouraged to collaborate independently of HSR&D assistance.

6. Evaluation Criteria

Proposals will be evaluated based on the extent to which they: 1) are innovative, but draw from the theoretical and empirical evidence base on organizational change and healthcare provider behavior; 2) have sound organizational structure; 3) involve key stakeholders in planning and execution; 4) show promise for enduring and expanding success throughout VHA; 5) rapidly create clinical and organizational improvement; 6) exhibit budget efficiency and network commitment, and 7) will add to the general scientific and practical understanding of implementation.

Because of the complexity of this process, funding will proceed in two phases.

6a. Phase 1: Planning Proposals

HSR&D will award up to $50,000 per planning application to fund up to six months of planning and application writing activities. These six months will allow applicants the necessary time to develop a sound research and dissemination plan, identify a clinical or organizational intervention, and generate support from all key stakeholders. These funds will support all aspects of planning and proposal writing (e.g. salary, travel, pilot data).

The planning award application should describe, in no more than two pages of 12 point font text, preliminary plans for addressing Objectives A and B above, involvement of researchers and key stakeholders, and the process to develop a final proposal. In addition, a budget and biosketches for Co-PIs should be included on VA forms 1313-2 to 1313-6. The planning award application also must contain a one page letter of support signed by the VISN director(s) and one additional page for optimal timeline, if desired.

Proposals will undergo peer review by an ad hoc committee, selected from members of the HSR&D Scientific Review and Evaluation Board, Network representatives and VHA Headquarters staff. Proposals will be evaluated on the basis of their scientific merit and expected contribution to improving VA health services. Proposals recommended for approval will then be considered for funding.

Planning award applications will be due October 15 and April 15 of every year, with funding correspondingly announced by December 15 and June 15. Note: for FY2004 only the submission deadlines will be November 15, 2003 and April 15, 2004. Funding will be announced by December 15, 2003 and June 15, 2004. HSR&D expects to fund 15-20 planning awards in FY04 and anticipates that about 50% of the planning awards will result in fully funded proposals.

6b. Phase 2: Final Proposals

Successful planning awards are expected to result in submission of a Phase 2, Final Proposal. The final proposal must follow the standard format and requirements for an HSR&D merit review application (for instructions, see www.hsrd.research.va.gov/for_researchers/funding/application/guidelines/ch3.cfm). However,

unlike a traditional application, the 25-page narrative should relate to Objectives A and B (under the Objectives for Projects section). The narrative must also describe the plan for an unbiased evaluation of the proposed work.

HSR&D will award up to $300,000 per year, per VISN, per research team application, for up to three years total. However, budgetary efficiency will be a review criterion. This award, similar to Service Directed (QUERI) Projects (SDPs), can be used to fund the evaluation, training, performance monitoring, and salaries for clinical champions and evaluation personnel. Although VISNs are not required to contribute resources, VISN interest and support (for which resource contributions are a key indicator) will be an important review criterion. The VISN resource contributions should be described in the budget and in a signed letter from the VISN director. Resource contributions may be actual expenditures and also resources such as equipment purchase or use, staff time, or other forms of in-kind support. Networks are advised that HSR&D anticipates extremely limited travel funds for this award and are encouraged to assign travel costs to a Network budget contribution.

Applications will undergo peer review by an ad hoc committee, selected from members of the HSR&D Scientific Review and Evaluation Board, Network representatives and VHA Headquarters staff. Proposals will be evaluated on the basis of their scientific merit and expected contribution to improving VA health services. Proposals recommended for approval based on merit will be considered for funding. Phase 2 Final Proposals will be due 6 months after the award of the planning awards, at the standard HSR&D merit review dates (June 15 and December 15 of each year). HSR&D anticipates funding five new applications each year.

7. Mailing Address

Proposals submitted under this initiative may be sent by regular mail or Federal Express, through the Associate Chief for Research and Development at a VISN facility.

HSR&D/VISN Implementation Collaborative
HSR&D Service (124)
Department of Veterans Affairs
810 Vermont Avenue, NW
Washington, DC 20420

8. Inquiries

For further information regarding this solicitation, contact Pauline Sieverding, PhD, JD, MPA (202.254.0249) or pauline.sieverding@hq.med.va.gov.

Nelda P. Wray, M.D., MPH
Chief Research and Development Officer
