# Supplementary material for: An organizational framework and strategic implementation for system-level change to enhance research-based practice: QUERI Series
Source: Implement Sci. 2008 May 29;3:30. doi: 10.1186/1748-5908-3-30 (PMC2430586; doi:10.1186/1748-5908-3-30)
Supplement: Additional file 4 — Special solicitation for Service-Directed Projects (SDP) on implementation of research into practice (2003). A special form of funding and study focus to encourage action-oriented improvement research. [file 1748-5908-3-30-S4.pdf]

OFFICE OF RESEARCH AND DEVELOPMENT
HEALTH SERVICES RESEARCH AND DEVELOPMENT SERVICE (HSR&D)
AND
OFFICE OF QUALITY AND PERFORMANCE (OQP)

PROGRAM ANNOUNCEMENT

Quality Enhancement Research Initiative (QUERI)

Special Solicitation for Service Directed Projects (SDP) on Implementation of Research into Practice

1) **Purpose.** The Office of Research and Development (ORD) and the Office of Quality and Performance (OQP) are interested in rapid and systematic implementation of clinical research findings and recommendations into routine practice within the Veterans Health Administration (VHA). In addition, ORD and OQP seek to enhance implementation processes by expanding the knowledge of quality enhancement and research implementation processes, barriers and facilitators. Proposals responsive to this solicitation will include efforts designed to address any of the following:

   a) Implement and evaluate specific programs and strategies to enhance VHA health care quality and outcomes through implementation of research findings and guidelines into routine practice.

   b) Develop evidence and insights regarding quality enhancement and practice improvement processes, barriers and facilitators within VHA.

   c) Facilitate development of infrastructure and mechanisms within VHA healthcare facilities, Veterans Integrated Service Networks (VISNs), and Central Office to support ongoing improvement in healthcare practices, quality and outcomes.

2) **Background.** VHA is devoting significant effort and resources to improve the quality and outcomes of health care services. The Quality Enhancement Research Initiative (QUERI) promotes the systematic use of evidence as the basis of clinical decision-

making to optimize patient outcomes and achieve ongoing system-wide quality enhancement.

Steps in the QUERI process are:

a) **Step 1** Identify high-risk/high volume diseases or problems.

b) **Step 2** Identify best practices.

c) **Step 3** Define existing practice patterns and outcomes across VA and current variation from best practices.

d) **Step 4** Identify and implement interventions (including performance criteria) to promote best practices.

e) **Step 5** Document that best practices improve outcomes.

f) **Step 6** Document that outcomes are associated with improved health-related quality of life (HRQOL).

The QUERI process is being applied to high-risk and/or highly prevalent diseases or conditions among veterans. Currently there are QUERI groups addressing Chronic Heart Failure, Colorectal Cancer, Diabetes, HIV/AIDS, Ischemic Heart Disease, Mental Health, Spinal Cord Injury, and Substance Use Disorders. Other conditions may be addressed in the future by QUERI. Particular emphases of the Service Directed Projects are QUERI Steps 4, 5 and 6.

3) **Coordination With QUERI.** Principal investigators are encouraged to integrate their work with the established goals of the QUERI program. More information about the QUERI process is available at http://www.hsrd.research.va.gov/research/queri/overview.cfm. Investigators are encouraged to coordinate projects with ongoing QUERI initiatives and strategic plans, as well. Contact information for Coordinators of each QUERI Center is available at http://www.hsrd.research.va.gov/research/queri/overview.cfm#committee.

4) **Coordination with VHA Leadership and Field.** Projects responsive to this solicitation are recognized to be at the intersection of clinical practice, quality improvement and traditional health services research. Projects typically require interaction with VACO, VISNs, or health systems and may include developing plans and mechanisms for the quality improvement activity to be undertaken by VISNs or health systems.

5) **Project Features.** Recommended components of Service Directed Project proposals and plans are described in the Appendix. Investigators are encouraged to incorporate these project features into the narrative of the proposal, as appropriate to the nature of the proposed work.

6) **Eligibility.** Principal Investigators and co-Principal Investigators must hold a VA appointment of at least 5/8 time in order to be eligible to apply. Questions about eligibility may be referred to the HSR&D Eligibility Coordinator, Caryn Cohen, at caryn.cohen@hq.med.va.gov.

7) **Funding.** Projects submitted in response to this solicitation may not exceed 2 years in duration. Time for clinicians may be contributed or included in the budget request, as required by the intervention. Applicants are reminded to adhere to ORD guidelines for allowable use of funds for specific items.

8) **Format and Submission Instructions.** The recommended SDP proposal format and instructions for proposal submission are the same as described in the VHA Handbook for Investigator Initiated Research (IIR), supplemented by the proposal format recommendations provided in the Appendix to this SDP solicitation. An overview of the application process and submission for IIR funding is available in the VHA Handbook on the web at http://www.va.gov/resdev/directive/1204-1hk.pdf and from the Research and Development Office at each facility. The SDP application process requires submission of a concept paper describing the project. After approval of the concept paper, the investigator may submit a proposal according to published guidelines.

Inquiries. Specific guidance regarding proposal development may be obtained from Linda McIvor, QUERI Health Science Specialist, at Linda.McIvor@hq.med.va.gov

Appendix 1

Instructions for SDP Concept Paper Development and Submission

Use the LOI Cover Page (Form 10-1313-13) for the Concept Paper, with required signatures; see www.hsrd.research.va.gov/for_researchers/funding/application/guidelines/ch2_a.cfm. The Concept Paper should be 2-5 pages in length (1.5 or double spaced, exclusive of references) and should contain the following information:

- Summarize the main objectives and specific clinical focus of the proposed project. Briefly describe (1) the clinical/quality issue(s) to be addressed (citing, as appropriate, data on the clinical condition's/problem's prevalence/incidence, mortality/morbidity, quality of life consequences, economic consequences, etc.) and (2) the evidence-based clinical recommendations/guidelines or other foundations for the improvement initiative.
- Describe the proposed quality improvement strategy or program, citing appropriate literature and evidence supporting the hypothesized effectiveness of the proposed quality improvement approach.
- Describe the research/evaluation design and methods to be used and the type(s) of analyses to be performed.
- List key personnel involved in the project, including key staff names, affiliations and the discipline or specialty of the PI and co-PI (if applicable) and other key project participants. Describe the PI and other project participants' past or current involvement in QUERI (e.g., member of a QUERI Coordinator Center or Executive Committee). Note: HSR&D encourages designation of a single PI; no project may have more than one co-PI.
- State the expected project duration and estimated total cost.

Send electronic copies of the Cover Page and Concept Paper to: Linda.McIvor@hq.med.va.gov. Mail the original, signed copy to:

QUERI Program Manager (124Q)
Health Services Research and Development Service
Department of Veterans Affairs
810 Vermont Avenue, N.W.
Washington, DC 20420

Proposal and Concept Paper Submission Dates

Full proposals in response to this solicitation are due on June 15 and December 15 of each year. Concept papers are due by COB Eastern Time on January 15, March 15, July 15 and September 15 of each year (or by COB on the first business day following these deadlines, if a deadline falls on a weekend or holiday). Concept paper feedback and notification of acceptance will be provided approximately four weeks after each

submission deadline. Accordingly, concept papers submitted by the January 15 and March 15 deadlines will be reviewed in time to permit submission of a full proposal for the June 15 deadline, while concept papers submitted by the July 15 and September 15 deadlines will be reviewed in time to permit submission of a full proposal for the December 15 deadline.

Please contact Linda McIvor (Linda.McIvor@hq.med.va.gov), QUERI Health Science Specialist, with any questions regarding concept paper instructions or submission dates.

Appendix 2

Instructions for SDP Proposal Development and Submission

The format of the SDP proposal is the same as described in the VHA Handbook for Investigator Initiated Research (IIR), which can be found at http://www.va.gov/resdev/directive/1204-1hk.pdf.

Because of the unique nature of SDP projects and quality improvement/implementation research, the following content areas should be addressed if applicable to the proposed project. The descriptive guidance provided below is intended to supplement and elaborate on the IIR guidelines and produce proposals specifically responsive to the SDP solicitation.

Proposal Narrative

The narrative is the core of the application and the focus of merit review. It should include comprehensive and detailed information about the rationale, goals and methods of the proposed project (including the project's intervention and evaluation components) to permit consideration of feasibility, value and appropriateness. Detailed information responsive to the criteria listed below should be provided in clear, concise, self-contained answers.

1) **Research Objectives (1 page)**

   a) **Overall project goal(s) and questions/hypotheses.** Briefly summarize the quality enhancement (QE) or performance problem(s) the project is targeting (e.g., lack of adherence to established best practice recommendations, excessive levels of practice variation, poor patient or system outcomes). Describe the project's overall goal(s), in light of these performance or quality problem(s). If the project aims to address an intervening barrier or factor *contributing* to the quality problem (but not *directly causing* the quality problem), explain how the project will contribute to solution of the overarching quality problem, and why a direct solution is not possible (at all or at this time).

   b) **Hypotheses.** Briefly state the key research or evaluation questions and hypotheses.

2) **Background of Context (2-3 pages)**

   a) **Previous published research.** Briefly summarize published research addressing the quality problem (including its magnitude and nature, its causes, its consequences, and efforts to address it) to provide a context for the proposed study. Do not provide details of previous studies if they are discussed later. Provide the reader with a general summary and orientation to the current "state

of the art" by indicating the types of research already completed, and the general findings and results.

b) **Current practices, determinants, barriers and facilitators.** Describe available evidence concerning (a) current and prevailing clinical practices within the site(s) participating in the project, and evidence regarding (b) barriers and facilitators to change, to provide appropriate background and context for the planned quality enhancement approach and for any primary data collection planned to further understand current practices, barriers and facilitators (as a preliminary step in finalizing intervention plans). Cite relevant evidence from QUERI research, other VA research and/or non-VA studies (including general literature discussing quality enhancement barriers and facilitators). Discuss the validity, relevance and limitations of this evidence. Describe the primary determinants of prevailing practices, using a "causal" or "logic" model diagram to depict hypothesized causal influences on these practices. Include individual, organizational and environmental factors hypothesized to influence these practices. Discuss barriers and facilitators to change, which are relevant to this model. Briefly highlight barriers and facilitators to be addressed by your quality enhancement intervention or program.

3) **Significance (1-2 pages)**

a) **Underlying quality enhancement/performance issue(s).** Provide (a) evidence documenting the existence and magnitude of the quality problem(s) to be addressed in the project, and (b) evidence and/or discussion of the quality problem's implications and impacts on quality of life, mortality and morbidity, economic issues, etc. Explain the importance of addressing the problem and the potential significance of the project. Comment on the policy, practice and research significance of the project.

b) **Clinical research finding, recommendation or best practice**. Describe the significance of the clinical recommendation(s), guideline(s) and/or best practice(s) to be implemented to address the quality gaps or problem. Describe the evidence base underlying the recommendations (including its strength, using standard strength-of-evidence criteria, and its origins), the consensus or other development (or evidence synthesis) process used in their creation and other attributes relevant to their expected acceptance and validity. Discuss potential points of controversy, lack of consensus or acceptance by clinicians or any other relevant stakeholder group, or likely appropriate (acceptable) variation or deviation from the recommendations.

4) **Methods (15 pages)**

a) **Intervention overview.** Describe the overall conceptual framework for your approach, citing specific sources and justifying the selection of the source(s) and framework for the specific quality problem and intervention approach you plan.

Clearly distinguish among the behavior or organizational change strategy (or “intervention”), the underlying clinical intervention(s) targeted in the project, and any new organizational arrangements (structures, policies, practices) to be implemented (via the behavior or organizational change intervention) to achieve the desired improvements and changes in clinical practices and quality.

b) **Intervention details.** Describe, as appropriate, each major component of the intervention (in each experimental arm, as applicable). Justify inclusion of each component by (a) explaining the applicable barrier(s) and facilitator(s) it addresses and (b) the specific purpose(s) and (c) the mechanisms of operation. Describe the operation of each component, to facilitate replication and reliable interpretation. Consider the use of a table to summarize the match between components and barriers and facilitators, by listing the major determinants of prevailing practices and barriers and facilitators to change in the table rows, and the intervention components in the table columns. Alternatively, consider use of the conceptual model (see “Current practices” section above) as a mechanism for clearly illustrating the purpose of each component. If applicable, describe intervention components that will perform the following functions:

1) communicate the legitimacy (e.g., evidence base) of recommended practices (to facilitate their acceptance by the target clinicians, managers, patients, and/or other stakeholders),
2) motivate clinician, manager, patient willingness to change via presentation of evidence of a quality/performance problem or via other means
3) establish, disseminate and reinforce professional (patient) norms favoring the recommended practices and countering current (non-adherent) practices and conveying the advantages of the preferred practices over current practices,
4) create or strengthen external expectations and interest in improved quality (among professional, policy, public, special interest, and/or other groups),
5) educate clinicians, staff, patients regarding desired/expected roles, practices and professional behaviors,
6) enhance clinician/staff/patient knowledge regarding the desired clinical practices and/or enhance skills in performing desired practices,
7) create conditions facilitating and favoring desired practices in routine care, including financial, administrative and other conditions,
8) redesign other aspects of the delivery system and organization to facilitate improved practices,
9) implement the desired care model or organizational arrangements and the behavior/organizational change efforts in a manner adapted to the target practice settings, to maximize success,
10) monitor and continually refine implementation of the new practices, including actions needed to remove any barriers that may arise.

c) **Justification of research design.** Describe and justify the overall research design, including issues such as the experimental unit (facility, clinic, team,

clinician, patient) and other major design features. Justify the choice of a randomized or non-randomized design by explicitly discussing one or more likely alternatives, their strengths and weaknesses, and the reasons for rejecting the alternatives in favor of the selected design.

d) **Variables, measures and data collection methods/plans.** Describe, in detail, variables and measures to be collected and protocols for collecting the relevant data. Consider use of a table to list all variables and measures, their relevance to study of research questions and hypotheses, data sources and data collection methods. Describe human subjects and IRB issues, secondary or administrative data issues (availability, validity, etc.) and other potential barriers to successful completion of data collection plans.

e) **Diagnostic analysis and study of practice determinants**. If relevant, provide detailed information on methods to study current practice patterns and their determinants. Perform a detailed diagnostic analysis of current practices, barriers and facilitators to further plan and refine the quality enhancement approach. Describe the motivation and aims for this data collection effort, details of the data to be collected and collection methods, and details of the analysis and interpretation methods.

f) **Formative evaluation: overall plan.** If applicable, describe plans for monitoring implementation and progress of the quality enhancement effort, and refining the effort based upon this ongoing monitoring. Justify decisions to attend to certain features of the implementation effort but not others, or to exclude formative evaluation altogether.

g) **Impact (summative) evaluation: overall plan.** Describe plans for measuring the quality enhancement program's impacts on key structures, processes and outcomes of interest. Address the estimated impact on organizational and care delivery structures, policies and practices and clinician knowledge, attitudes and behaviors, outcomes such as patient functional, clinical and psychosocial outcomes, clinician satisfaction, economic outcomes or other system features. Refer to the research questions and conceptual model to identify specific structures, processes and outcomes of interest that will be enhanced.

h) **Subjects and subject recruitment.** Describe plans for identifying and recruiting all relevant participants, including clinicians, other staff (managers, support staff), patients, patient family members or caregivers, etc. Address any human subjects issues not already discussed.

i) **Project sites and site recruitment.** List and describe the participating sites and the process(es) used in recruiting these (or additional) sites. If site recruitment is not yet complete, provide evidence supporting the projected success of recruitment efforts, along with proposed timeline.

j) **Randomization protocol.** As applicable, describe methods for randomizing sites, providers, patients and/or other experimental units. Justify the selection of the unit of randomization and randomization protocol, including any tradeoffs or alternatives (and reasons for rejecting these alternatives in favor of the selected approach).

k) **Usual care condition.** Describe, as appropriate, any control, comparison or "usual care" condition included in your project, including any intervention or other activities to be directed at the comparison sites.

l) **Assurance of patient safety.** Describe how patient safety will be assured. Systems must be in place to assure patient safety, including systems for vigilant monitoring and ongoing feedback. Redundant systems should be in place to ensure that standard quality practice is maintained in context of the proposed intervention.

m) **Sustainability**. Evaluate and propose plans for sustainability of the new care processes and quality, if successfully implemented. Include specific actions or activities planned for phases of the effort, i.e., from initial planning to the post-project phase. Discuss projected plans for "regional roll-out" or "national roll-out" follow-up to the project (if the project represents an initial demonstration project), including planned activities to facilitate initiation and success of such follow-up.

n) **Analysis plans and methods.** Describe general analytical approaches for quantitative and qualitative data to be collected as part of the diagnostic analysis, formative evaluation and impact/summative evaluation. Present sample size/power calculations for relevant quantitative analyses. Present illustrative statistical models and qualitative data analysis methods, as appropriate. Discuss sensitivity analyses, validation efforts and other plans to enhance confidence in results. Discuss plans for interpreting results in terms of their clinical, practice and policy significance and implications for theory and research, within and outside VHA.

5) **Project management plan (1-2 pages)**

a) **Intervention management plans.** Describe key intervention activities in a combined intervention/evaluation timeline (Gantt Chart). Describe, in detail, plans for managing and staffing the intervention component of the project.

b) **Evaluation management plans.** Describe key evaluation activities in the combined intervention/evaluation timeline (Gantt Chart). Describe plans for managing and staffing the evaluation component of the project. Explain any overlap between intervention and evaluation team membership and explain plans to minimize bias due to this overlap.

c) **Team qualifications.** Describe the roles and responsibilities of each member of the intervention and evaluation teams. *Intervention team:* Describe the team's relevant expertise in areas such as healthcare management and organizational change, translation or research into practice/knowledge utilization within clinical settings. In addition, briefly summarize pertinent individual PI and investigator experience and expertise in areas such as (a) clinical policy development, (b) clinical leadership and (c) practice improvement efforts. This experience/expertise may include involvement in VHA CO-level activities, VISN-level activities, facility-levels activities and non-VHA activities (non-VA healthcare delivery settings, other government settings, specialty society activities, etc.). Describe experience only if directly relevant to the proposed project. *Evaluation team:* Describe the team's relevant expertise in areas such as management and organizational research, evaluation of interventions to implement evidence-based clinical practice, healthcare quality and outcomes assessment, and other relevant areas. Describe experience in use of the specific analytical approaches and methods proposed for the project.
